# A Robust Approach to Enhance Tumor-selective Accumulation of Nanoparticles

**DOI:** 10.18632/oncotarget.227

**Published:** 2011-03-01

**Authors:** Yuan Qiao, Xin Huang, Sridhar Nimmagadda, Renyuan Bai, Verena Staedtke, Catherine A. Foss, Ian Cheong, Matthias Holdhoff, Yoshinori Kato, Martin G. Pomper, Gregory J. Riggins, Kenneth W. Kinzler, Luis A. Diaz, Bert Vogelstein, Shibin Zhou

**Affiliations:** ^1^ The Ludwig Center for Cancer Genetics and Therapeutics, Howard Hughes Medical Institute and Sidney Kimmel Cancer Center at the Johns Hopkins Medical Institutions, Baltimore, Maryland 21231, USA; ^2^ Russell H. Morgan Department of Radiology and Radiological Sciences, Johns Hopkins Medical Institutions, Baltimore, Maryland 21231, USA; ^3^ Department of Neurosurgery, Johns Hopkins Medical Institutions, Baltimore, Maryland 21231, USA

**Keywords:** Tumor, Vasculature, Nanoparticle, Inflammation, TNF-α

## Abstract

While nanoparticles have shown great promise as drug carriers in cancer therapy, their effectiveness is critically dependent on the structural characteristics of the tumor vasculature. Here we demonstrate that several agents capable of inducing vascular responses akin to those observed in inflammatory processes enhance the accumulation of nanoparticles in tumors. The vascular-active agents tested in this study included a bacterium, a pro-inflammatory cytokine, and microtubule-destabilizing drugs. Using radiolabeled nanoparticles, we show that such agents can increase the tumor to blood ratio of radioactivity by more than 20-fold compared to nanoparticles alone. Moreover, vascular-active agents dramatically improved the therapeutic effect of nanoparticles containing radioactive isotopes or chemotherapeutic agents. This resulted in cures of animals with subcutaneous tumors and significantly prolonged the survival of animals with orthotopic brain tumors. In principle, a variety of vascular-active agents and macromolecular anticancer formulations can be combined, which makes this approach broadly applicable and particularly suited for the treatment of patients who have failed standard therapies.

## INTRODUCTION

Wounding results in increased vascular permeability, a process that is markedly enhanced if a wound becomes infected. In response to infection, the mammalian host mobilizes an army of immunoglobulins, complement, white blood cells and cytokines. To allow this army to engage the enemy, the vascular system at the site of infection must open its gates. This process has been studied in detail and many of the biochemical mechanisms have been identified[1].

Interestingly, it has been said that tumors resemble “unhealed wounds”[2]. Accordingly, it is known that the vasculature of tumors is different from that of normal cells, and much effort has gone into exploiting this difference through therapeutic agents like Avastin[3-5]. A particularly important phenomenon related to this vascular distinction is referred to as Enhanced Permeability and Retention (EPR)[6]. EPR has been identified in many experimental tumor systems and is believed to result from the aberrant tumor vasculature combined with a lack of functional lymphatics in solid tumors. Because of its selectivity for large molecules, EPR has been exploited for therapeutic purposes by using macromolecular drugs or nanoparticles within an appropriate size range[7-10]. One notable example is Doxil, a liposomal formulation of doxorubicin, which has been approved for the treatment of human cancers.

We wondered whether generating an inflammatory environment within a tumor could enhance the EPR effect, just as infection within a wound dramatically enhances its vascular permeability. To test this approach, we determined whether inflammatory and vascular-active agents could improve selective nanoparticle accumulation within tumors and whether such accumulation would lead to improved therapeutic results in animal models.

## RESULTS

### Bacterial infection enhances antibody accumulation in experimental tumors

The research described in this work was stimulated by unexpected observations made through the investigation of *C. novyi-NT*, an attenuated anaerobic bacterial strain that can infect experimental tumors[11]. This infection often leads to eradication of the internal hypoxic regions of tumors but leaves the oxygenated rim of the tumors intact. *C. novyi-NT* secretes an enzyme called liposomase at high levels in the infected tumors[12, 13]. We hypothesized that a radiolabeled anti-liposomase antibody would synergize with *C. novyi-NT* by binding to liposomase secreted by the bacteria, thereby eradicating the oxygenated tumor rim through β-particle irradiation. A monoclonal antibody against liposomase was generated and used to evaluate this hypothesis (see Methods).

**Figure 1:**
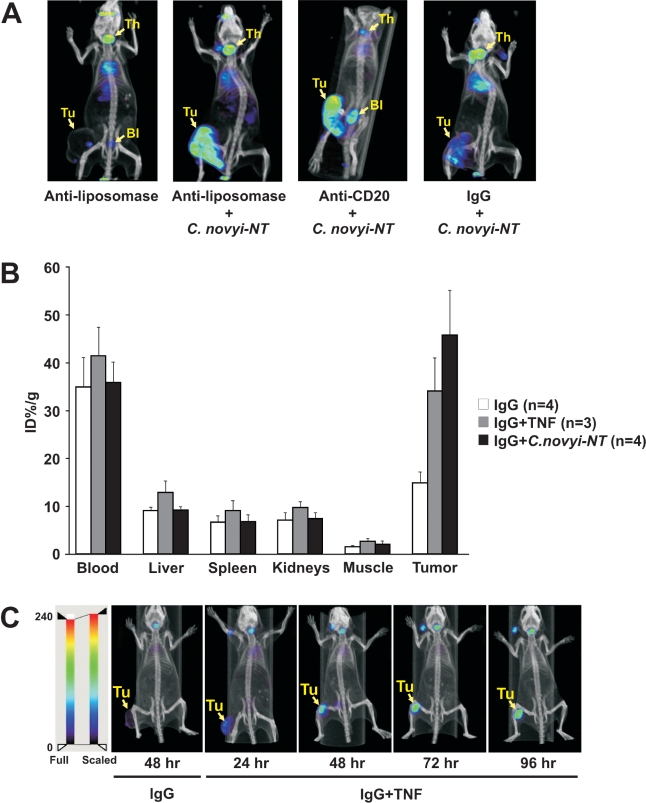
Inflammatory responses enhance tumor-selective accumulation of radiolabeled antibodies (A) BALB/c mice bearing subcutaneous CT26 tumors were administered *C. novyi-NT* spores plus ^125^I-labeled liposomase antibody, CD20 antibody, or an IgG control antibody by tail vein injection. The animals were imaged by SPECT/CT 24 hours thereafter. Tumor (Tu), thyroid (Th) and bladder (Bl) are indicated. (B, C) Tumor-bearing mice were administered ^125^I-labeled IgG plus *C. novyi-NT* spores or TNF-α by tail vein injection. For biodistribution analysis (B), mice were sacrificed 48 hours later and percent injected dose per gram of tissue (ID%/g) was determined. Means and s.d. are shown. For imaging study (C), SPECT/CT images were taken at the indicated time points after the injections. Tumor (Tu) is indicated.

Mice bearing subcutaneous CT26 tumors were intraveneously injected with *C. novyi-NT* spores together with the radiolabeled anti-liposomase antibody or with a similarly labeled IgG control antibody. The anti-liposomase antibody was highly enriched in the tumors infected with *C. novyi-NT* but not in uninfected tumors (Fig. 1A). Surprisingly, however, the radiolabeled IgG control antibody was also enriched in the *C. novyi-NT* infected tumors, albeit to a lesser extent (Fig. 1A). Biodistribution analyses showed that the level of radioactivity in the tumor was four-fold higher than that in most normal tissues (Fig. 1B).

To further confirm that the accumulation in the tumors was not antibody-specific, we repeated the experiment with another antibody generated against human CD20, a B-cell antigen. The partially humanized version of this antibody, Rituximab, has been marketed for the treatment of B cell lymphoma and chronic lymphocytic leukemia[14, 15]. Systemically administered anti-CD20 antibody was also enriched in the tumor if the animal was simultaneously injected with *C. novyi-NT* spores (Fig. 1A).

### Bacterial infection and pro-inflammatory cytokine both enhance tumor-selective accumulation of macromolecular drug formulations

We reasoned that the inflammatory response to the bacterial infection led to an increased vascular permeability, resulting in the preferential antibody accumulation at the infected tumor site. We therefore sought to identify a pro-inflammatory cytokine that might mimic the effects of *C. novyi-NT*. Among those considered, Tumor Necrosis Factor-α (TNF-α) was of particular interest as this cytokine has been identified as the serum factor responsible for endotoxin-induced vascular permeabilization[16, 17]. Furthermore, a similar hemorrhagic necrosis in tumors is observed following systemic administration of either TNF-α or *C. novyi-NT* spores[11, 16]. Based on these parallels, we repeated the protocol described above, substituting systemically-administered TNF-α for *C. novyi-NT* spores. When CT26 tumor-bearing mice were injected with murine TNF-α and radiolabeled murine IgG, significant IgG accumulation was observed in the tumors but not in the normal tissues (Fig. 1B and C). A time course study revealed that the IgG tumor accumulation progressed slowly and peaked between 72 and 96 hours after injection (Fig. 1C).

The effect of vascular-active agents on tumor vasculature will henceforth be referred to as Enhanced EPR (E^2^PR). Sterically stabilized liposomal nanoparticles (SSLs) of ~100 nm in diameter have been shown to be susceptible to the EPR effect[8]. To evaluate whether such liposomes were susceptible to E^2^PR, we fabricated radioactive liposomes using a Bolton-Hunter (BH) reagent-based iodination strategy[18]. Iodinated BH reagent labels proteins by forming amide bonds with free amino groups such as those present on arginine[19]. SSLs were loaded with arginine at low pH and then the loaded SSLs were incubated with ^125^I-labeled BH reagent. The ^125^I-BH reagent passed through the lipid bilayer but was unable to exit after covalent binding to the arginine because of the latter's positive charge. We were thus able to achieve a very high concentration of radioactivity within the SSLs while avoiding prolonged exposure to the radioactivity during the preparation.

**Figure 2:**
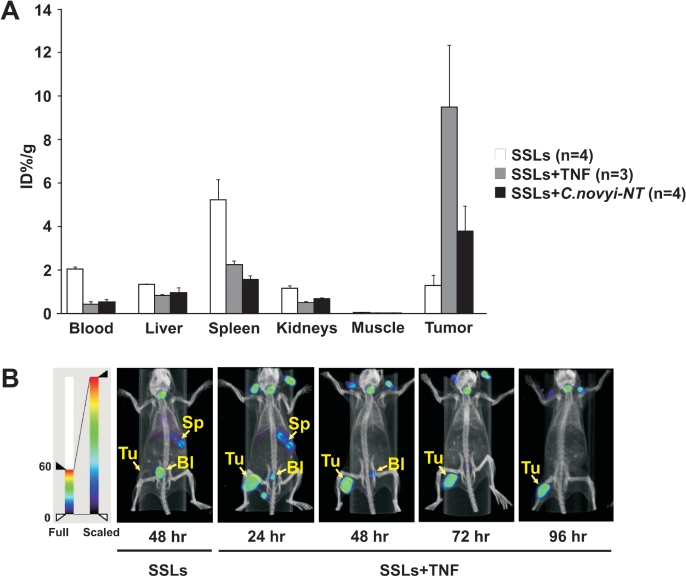
Inflammatory responses enhance tumor-selective accumulation of radiolabeled SSLs BALB/c mice bearing subcutaneous CT26 tumors were administered ^125^I-labeled SSLs plus *C. novyi-NT* spores or TNF-α by tail vein injection. For biodistribution (A), mice were sacrificed 48 hours later and percent injected dose per gram of tissue (ID%/g) was determined. Means and s.d. are shown. For imaging analysis (B), SPECT/CT images were taken at the indicated time points after the injections. Tumor (Tu), bladder (Bl) and spleen (Sp) are indicated.

^125^I-labeled SSLs were intravenously injected into tumor-bearing mice in combination with either *C. novyi-NT* or TNF-α. Both *C. novyi-NT* and TNF-α treatments significantly augmented the selective retention of ^125^I within tumors (Fig. 2). Furthermore, the radioactivity in normal tissues was markedly lower compared to the animals treated with ^125^I-labeled SSLs without TNF-α or *C. novyi-NT* (Fig. 2A). Thus, the tumor-to-blood ratio of radioactivity following TNF-α treatment was as high as 22-fold, far higher than achieved with radiolabeled IgG (compare Fig. 2A to Fig. 1B). SPECT/CT also revealed that the kinetics of tumor accumulation was different with radiolabeled SSLs than with IgG: SSL accumulation peaked at 24 hours, 48 - 72 hours earlier than IgG.

Like EPR, the effect of E^2^PR is particle size-dependent. In contrast to ^125^I-labeled SSLs, tumor retention of ^125^I-labeled arginine (the substrate of ^125^I labeling in SSLs) is not affected by TNF-α. However, at the other end of the size spectrum, ^125^I-labeled *C. novyi-NT* spores (~1 μm in diameter[20]) are highly enriched in tumors only when combined with TNF-α (data not shown). Thus, E^2^PR appears to reflect a more substantial vascular disruption than EPR: while EPR favors accumulation of nanoparticles in the range around 100 nm[8], E^2^PR extends that range to >;1 μm.

To determine whether the accumulation was dependent on the volume of the tumor, we injected TNF-α plus ^125^I-labeled IgG or ^125^I-labeled SSLs into animals with a small subcutaneous tumor on one flank and a large tumor on the other flank. SPECT/CT showed retention of radioactivity in both tumors (examples in Supplementary Fig. S1). We also tested the relative timing of injection of TNF-α and ^125^I-labeled SSLs. Though TNF-α and SSLs were administered jointly in the experiments recorded above, we found that similar results were obtained when TNF-α was administered within12 hours after SSLs. Conversely, E^2^PR was not observed when TNF-α was administered 6 hours prior to SSL administration (data not shown).

Microtubule-interacting agents are also able to disrupt the tumor vasculature[21]. We therefore determined whether such agents could induce E^2^PR. Combretastatin A4P (CA4P) and vinorelbine are microtubule-interacting agents with completely different structures and modes of interaction with microtubules[22, 23]. Injection of either resulted in E^2^PR, though not as impressively as TNF-α (Supplementary Fig. S2).

### TNF-α and macromolecular drug formulations synergize in the treatment of experimental tumors

**Figure 3:**
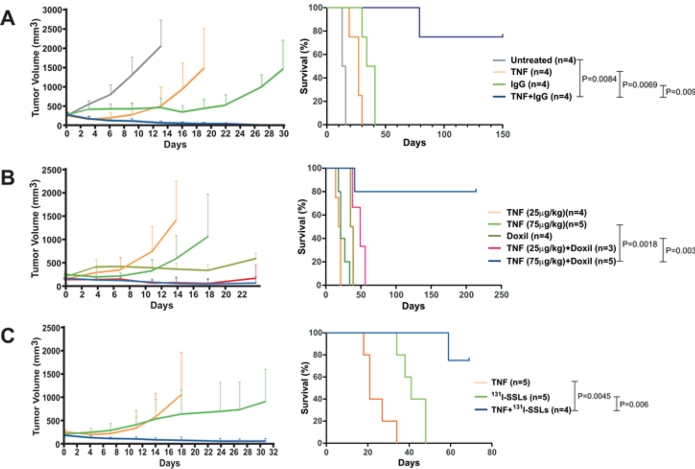
TNF-α enhances the antitumor activity of macromolecular drug formulations Tumor-bearing mice were treated on day 0 with a single dose of the combinations of TNF-α plus ^131^I-labeled IgG (A), Doxil (B), or ^131^I-labeled SSLs (C), respectively. The therapeutic effects on tumor volume and animal survival are shown. Means and s.e.m. are illustrated. The number of animals used in each experimental arm is shown in parentheses. P values between arms are also shown.

We next investigated whether the E^2^PR could be translated into therapeutic gain. Mice bearing fully developed CT26 tumors were treated by simultaneous i.v. injections of TNF-α plus Doxil (10 mg/kg) or radiolabeled IgG. ^131^I rather than ^125^I was chosen for radiolabeling in light of the type of ionizing radiation required for a radiotherapeutic effect. Although treatment with Doxil or ^131^I-labeled IgG in the absence of TNF-α retarded tumor growth and prolonged animal survival, the tumors always grew back (Fig. 3A and B). When combined with TNF-α, however, a single administration of these agents led to complete tumor regression in all animals and long-term cures in more than 75% of them. When a lower dose (25 μg/kg) of TNF-α was used, none of the treated animals were cured, although prolonged survival was observed. It is important to note that humans tolerate multiple injections (3 infusions/week) of a dose comparable to the highest dose of TNF-α we used[24]. We also tested SSLs containing ^131^I, generated using the chemical trapping approach described above. While ^131^I-labeled SSLs alone retarded tumor growth, complete tumor regression and cures were only observed when they were used in combination with TNF-α (Fig. 3C).

**Figure 4:**
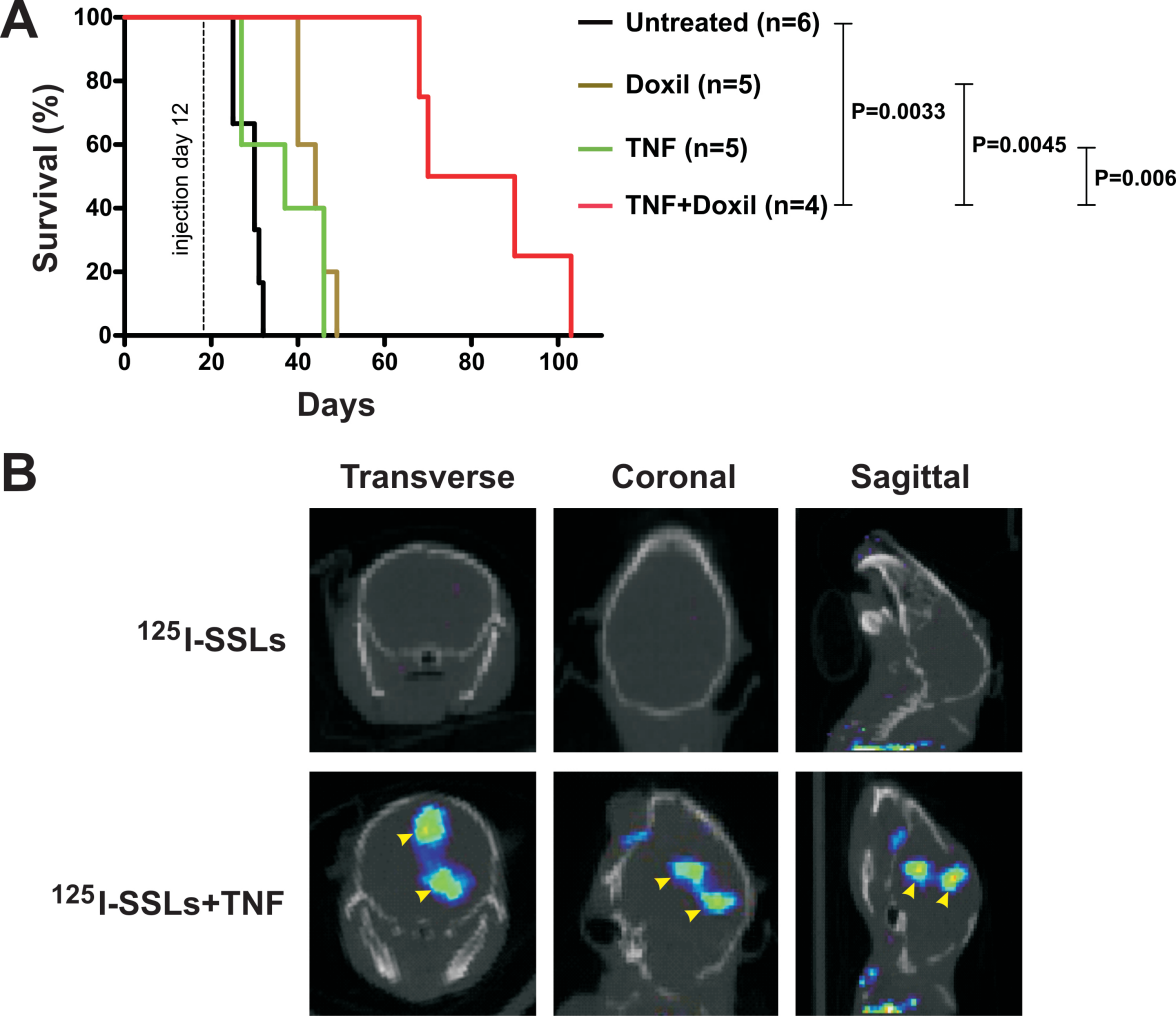
Vascular effect of TNF-α on a brain tumor model (A) C57BL6 mice bearing orthotopic brain tumors were treated with a single dose of the indicated therapeutic agents 12 days after tumor implantation. The number of animals used in each experimental arm and P values between arms are shown. (B) SPECT-CT images were obtained 48 hours following the indicated treatments, which were performed 25 days following tumor implantation. Transverse, coronal, and sagittal images are shown and tumors indicated by the arrowheads. In this particular animal, two tumor nodules developed along the injection track and both showed tumor accumulation of ^125^I-labeled SSLs when TNF-α was co-administered.

Finally, we evaluated the therapeutic potential of E^2^PR in a murine model of glioblastoma multiforme (GBM). When implanted orthotopically, the brain tumor cell line GL261 forms very aggressive tumors, killing animals within about a month (Fig. 4A). At the histologic level, these tumors are very similar to human GBM, manifesting an infiltrative growth pattern, necrosis and neovascularization[25]. Following stereotactic injection of GL261 cells into the frontal lobe, brain tumors were allowed to grow to substantial size, then ^125^I-labeled SSLs with or without TNF-α were administered. Tumor accumulation of the radiolabeled SSLs was only observed in TNF-α treated animals (Fig. 4B). Mice with similar tumors were injected with Doxil, either with or without TNF-α. The combination clearly had a therapeutic benefit, prolonging survival up to 103 days even in this highly challenging pre-clinical model (Fig. 4A). Both Doxil and TNF-α showed limited therapeutic benefit when used as single agents, with no animal surviving beyond 50 days following tumor implantation.

## DISCUSSION

The major limitation for most chemotherapeutic agents is their toxicity toward normal tissues, which prohibits the use of doses high enough to eradicate all cancer cells. One approach to address this problem is to develop agents that are delivered to all cells but are preferentially toxic to tumor cells because of the abnormal signaling pathways. This strategy underlies the success of agents such as Gleevec (imatinib) and Iressa (gefitinib)[26, 27]. A second approach is to use agents that bind to extracellular molecules present at higher concentrations on the surface of tumor cells, such as Herceptin (trastuzumab) and Erbitux (cetuximab)[28, 29]. The third approach takes advantage of the abnormal vasculature present in tumors, allowing preferential accumulation of nanoparticles (the EPR effect)[6, 30]. Though all approaches have merit, the third has the advantage that virtually any drug, including a wealth of clinically approved agents, can in theory be made more effective by their incorporation into nanoparticles of appropriate sizes. The ability to use agents that are already clinically approved poses many practical advantages with respect to the performance of clinical trials and the duration of the drug approval process.

In this work, we have attempted to enhance the third approach through pharmacologic manipulation of the abnormal vasculature already present in tumors. We show that E^2^PR can dramatically increase the tumor:blood ratio of nanoparticles, as assessed by biodistribution, and we hypothesize that this increase is primarily responsible for the enhanced therapeutic response. It is worth noting that even a small difference in the intratumoral concentration of an agent can make a large difference in therapeutic effect[31]. In the studies described here, E^2^PR led to a tumor:blood ratio of more than 22-fold (Fig. 2A). However, it should be noted that TNF-α, *C. novyi-NT* infection, and doxorubicin-induced cancer cell apoptosis are all able to induce or enhance a host immune response that could have contributed to the observed therapeutic response.

We were particularly encouraged with the results in the GBM model. This tumor type in humans is highly recalcitrant to conventional therapies, leading to a dismal prognosis for patients with this disease. The blood-brain barrier is at least partly to blame for the limited efficacy of chemotherapy[32]. We found that TNF-α treatment could help breach the blood-brain barrier and result in major accumulations of ^125^I-labeled SSLs in the orthotopically implanted brain tumors as well as significantly prolong the survival of the tumor-bearing animals (Fig. 4). As the mouse cranial cavity is small, murine brain tumors are particularly difficult to treat as even a minimal amount of growth of a pre-existing tumor is lethal.

In sum, our results suggest a way to improve the therapeutic efficacy of conventional and novel drugs by incorporating them into nanoparticles and injecting them together with vascular-active agents such as TNF-α. The approach is versatile, as it should be practicable with a variety of nanoparticle formulations as well as with diverse chemical and radioactive agents. Different classes of agents with E^2^PR effect can be employed to enhance their tumor accumulation. In addition to those tested in this work, other vasoactive agents, including vascular endothelial growth factor (VEGF), are likely to have similar effect. An imaging-based companion test may be developed in the future to assess which vasoactive agent is most effective, as the vasculature of individual cancers may have varied sensitivity to a specific vascular-active agent. Future clinical trials can address whether this strategy is as efficacious in humans as it is in mice. To accelerate the translation of this approach, we have intentionally used TNF-α at doses either below or comparable to the maximum tolerated dose (MTD) as defined in a number of clinical trials [33].

## MATERIALS AND METHODS

### Cell Lines

CT26 (CRL-2638) murine colorectal adenocarcinoma cells were purchased from the American Type Culture Collection (ATCC) and grown in McCoy's 5A Medium (Invitrogen) supplemented with 10% Fetal Bovine Serum (FBS, HyClone) at 37°C with 5% CO_2_. GL261 glioma cells were kindly provided by Dr. Michael Lim (Johns Hopkins University, Baltimore) and maintained in DMEM media (ATCC) supplemented with 10% FBS.

### Reagents

Bolton-Hunter reagent (BH, N-succinimidyl-3-(4-hydoxyphenyl)-propionate) and TNF-α (mouse, recombinant) were purchased from Sigma-Aldrich. Radioiodines (Sodium 125- or 131-iodide) were purchased from MP Biomedicals and Nordion, respectively. IODO-GEN was purchased from Pierce. Mouse monoclonal IgG1 isotype control antibody (ab18447) and CD20 antibody (ab8237) were purchased from Abcam. PEGylated liposomal doxorubicin (DOXIL®) was purchased from Tibotec Therapeutics. Hydrogenated Chicken Egg L-α-Phosphatidylcholine (HEPC), 1,2-Distearoyl-sn-Glycero-3-Phosphoethanolamine-N-[Methoxy(Polyethylene glycol)-2000] (DSPE-PEG2000) and Cholesterol (Chol) were purchased from Avanti Polar Lipids. *C. novyi-NT* spores were prepared as previously described [11].

### Animal models

All animal experiments were overseen and approved by the Animal Welfare Committee of Johns Hopkins University, and were in compliance with the University standards. For the subcutaneous tumor model, female, six to eight week-old BALB/c mice (Harlan Breeders, ~20 g in weight) were used. Five million CT26 cells were injected subcutaneously into the right flank of each mouse and allowed to grow for ~10 days before randomization, group assignment, and treatment. *C. novyi-NT* spores were administered by a bolus tail vein injection of 300 million spores suspended in 0.2 mL of phosphate buffered saline, pH 7.5 (PBS). Cytotoxic anticancer agents were administered 16 hours later via the same route. TNF-α was reconstituted freshly before administration in doubly-distilled H2O at 100 μg/mL and diluted into 0.1% (w/v) BSA in PBS at a final concentration of 10 μg/mL. Cytotoxic agents were injected within a few minutes thereafter. Tumor volume was calculated as length × width^2^ × 0.5. For the orthotopic brain tumor model, female C57BL6 mice, 5-6 weeks of age, were purchased from the NCI-Frederick. Mice were anesthetized via intraperitoneal injection of 60 μL of a stock solution containing ketamine hydrochloride (75 mg/kg, Abbot Laboratories), xylazine (Xyla-ject®, 7.5 mg/kg, Phoenix Pharmaceutical), and ethanol (14.25%) in a sterile 0.9% NaCl solution. Following a 1-cm midline scalp incision, a 1-mm burr hole was placed over the right frontal bone, with its center 2 mm lateral to the sagittal suture and 1 mm anterior to the coronal suture. On a stereotactic frame, a sterile needle loaded with 20,000 GL261 cells was placed at a depth of 3 mm below the dura and the cells were injected slowly at a rate of 1 μL/minute. Afterwards, the animal was removed from the frame and the scalp incision closed with surgical staples. On day 12 post implantation of the tumor cells, a significant tumor was formed and 1 μg of mouse recombinant TNF-α or 100 μL of Doxil at 20 mg/kg, or both, were administered intravenously through the tail vein. Animals were monitored for potential side effects following drug administration. Animals were observed daily for any signs of deterioration, neurotoxicity, or movement disorders. They were inspected for signs of pain and distress, as per the Johns Hopkins Animal Care and Use Guidelines. If the symptoms persisted and resulted in debilitation, the moribund animals were euthanized. The brain and other organs were dissected and placed in formalin for further pathological studies. A single dose was administered for all therapeutic agents described above.

### Liposomase Antibody

Three peptides (JHU009A: CNVDLQQKLIEN; JHU009B: CYPEWGTKDENGNIRK; JHU009C: CDMAQMLRNLPVTE) were used to immunize the mice for generating antibodies against *C. novyi-NT* liposomase (A&G Pharmaceutical). After screening ~500 hybridoma clones by ELISA, one clone (JHU009-5F5) specific to the JHU009C peptide was eventually selected for the imaging study. The affinity and specificity of the JHU009-5F5 mAb were also confirmed by both ELISA and western blot analyses against purified liposomase protein [12].

### Radioiodination of Antibodies

Typically, 20 μg of purified antibody in 100 μL of PBS was added to an iodogen-coated vial. Sodium 125- or 131-iodide was then added to the vial at 2 to 5 mCi in 2 to 5 μL of 0.1 M NaOH, pH 10. The reaction was then incubated for 10 minutes at room temperature before purification on a PBS-equilibrated Sephadex G-25 desalting column (Amersham Biosciences) to remove unincorporated radioiodine. The radiochemical yield was typically 30% to 40%. The radiochemical purity was at least 95% as determined by thin-layer chromatography. Antibodies were labeled within 24 hours of use and stored in PBS at 4°C after labeling and purification.

### Preparation of Liposomes

A mixture of HEPC:Chol:DSPE-PEG2000 at a molar ratio of 50:45:5 was solubilized in chloroform, followed by drying to a thin film under rotary evaporation and then under vacuum for 2 hours. The film was hydrated with arginine solution (80 mmol/L) in 4-(2-hydroxyethyl)-piperazine-1-sulphonic acid (HEPES, 80 mmol/L, pH 8.0) and submerged in a 65°C sonication bath (Bransonic) to form Large Multilamellar Vesicles (MLVs). This lipid suspension was extruded 10 times through a double stack of 0.1 μm Nuclepore filters (Whatman) using a Lipex Thermobarrel Extruder (Northern Lipids). The resulting colloidal suspension of Single Unilamellar Vesicles (SUV) was dialyzed against 150 mmol/L phosphate buffer (pH 5.6) at 4°C to exchange the external milieu of the liposomes and then filter-sterilized. The mean size of the SUVs was ~100 nm in diameter and polydispersity index ~0.1 as determined by quasi-elastic light scattering using a Malvern Zetasizer 3000 (Malvern).

### Radioiodination of Bolton-Hunter reagent

Bolton-Hunter reagent (BH, N-hydroxysuccinimide (NHS) ester of HPPA) was labeled with sodium 125- or 131-iodide by the chloramine-T method and purified by solvent extraction. Briefly, 50 μL of chloramine T (4 mg/mL in phosphate buffer) and 3.7 to 37 MBq (0.1–1.0 mCi) of ^125^I-NaI or ^131^I-NaI were added to 2 μL of BH freshly solubilized in anhydrous dioxin (0.5 mg/mL). Iodination was achieved by incubation at room temperature for approximately 15 sec and then 400 μL of 100 mmol/L phosphate buffer (pH 7.4) was added. The radiolabeled BH was immediately extracted with 500 μL of toluene and the organic phase was removed and transferred to a glass tube. For the encapsulation of the reagent into liposomes, the organic solvent was evaporated using a dry nitrogen stream before adding the liposome suspension.

### Encapsulation of the Iodinated Reagents into the Liposomes

For the chemical entrapment of the iodinated BH, arginine-containing liposomes were incubated for 30 min at 37°C with ^125^I-BH. The labeling efficiency was determined by counting the liposome suspension before and after chromatography on a PD-10 column (GE Healthcare)[18]. The radiochemical yield was typically 50% to 70%.

### Biodistribution Assay

CT26-bearing BALB/c mice were injected via the tail vein with 50 μCi of ^125^I-liposomes or ^125^I-IgG1. Three to four mice in each experimental arm were sacrificed by cervical dislocation at 48 hours post injection. The liver, spleen, kidneys, muscle, and tumor were quickly removed as was ~0.1 mL of blood. The organs and blood were weighed and their radioactivity was measured with an automated gamma counter (1282 Compugamma CS, Pharmacia/LKB Nuclear). The percent injected dose per gram of tissue (ID%/g) was calculated by comparison with samples of a standard dilution of the initial dose. All measurements were corrected for decay.

### SPECT-CT Imaging

BALB/c mice bearing subcutaneous CT26 tumor or C57BL6 mice bearing orthotopic GL261 brain tumor were injected intravenously with 37.5 MBq (1 mCi) of either ^125^I-IgG1 or ^125^I-SSLs in saline. The mice were positioned on the X-SPECT (Gamma Medica-Ideas) gantry and scanned using two low-energy, high resolution pinhole collimators (Gamma Medica-Ideas) rotating through 360° in 6° increments for 40 seconds per increment. Immediately following SPECT acquisition, the mice were scanned by CT (X-SPECT) over a 4.6 cm field of view using a 600 mA, 50 kV beam. Data were reconstructed using the ordered subsets-expectation maximization algorithm. The SPECT and CT data were then coregistered using the instrument supplied software and displayed using AMIDE (http://amide.sourceforge.net/) or Amira software (Visage Imaging).

### Statistical Analysis

The statistical significance of percent survival between different experimental arms was determined by Long-rank analysis.

## SUPPLEMENTAL FIGURES




