# Supplementary figures and images for: A Robust Approach to Enhance Tumor-selective Accumulation of Nanoparticles
Source: Oncotarget. 2011 Mar 1;2(1-2):59–68. doi: 10.18632/oncotarget.227 (PMC3069713; doi:10.18632/oncotarget.227)

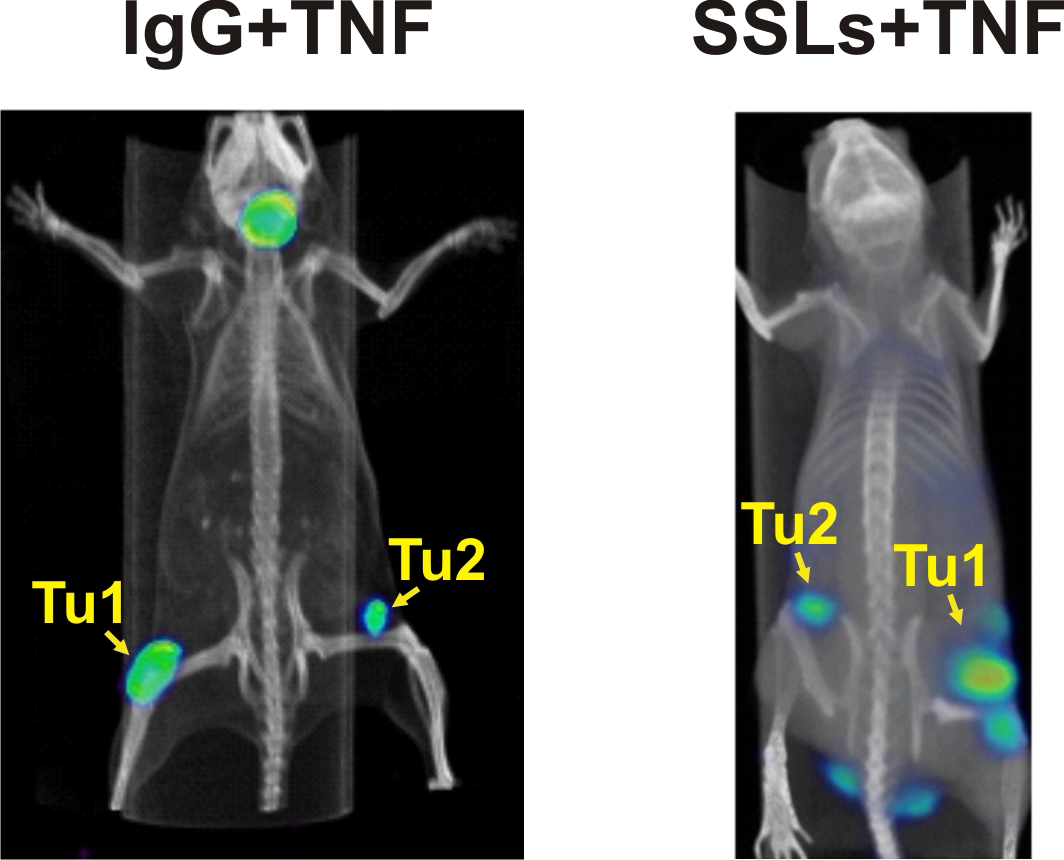

Supplement: Supplementary file 1 [file oncotarget-02-059-s001.tif]

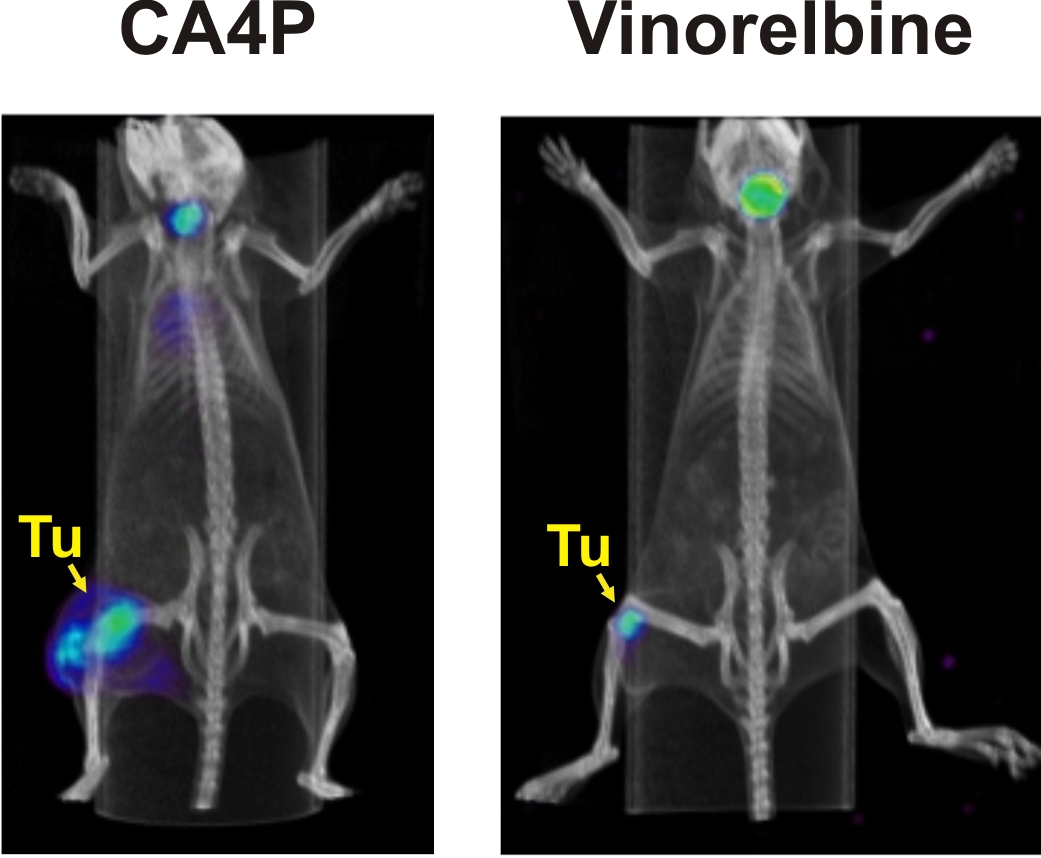

Supplement: Supplementary file 2 [file oncotarget-02-059-s002.tif]
